# 

**Published:** 1889-10

**Authors:** 


THE SOUTHERN SURGICAL AND GYNECOLOGICAL ASSOCIATION will be held in Nashville, Tenn., Nov. 12th, 13th and 14th, 1889.

President, Hunter McGuire, M. D., LL. D., Richmond, Virginia.

Chairman of the committee of arrangements, Duncan Eve, M. D , Nashville, Tennessee.

Numerous interesting papers to be read.

Members of the medical profession are cordially invited to attend.

